# Strategies to overcome DC dysregulation in the tumor microenvironment

**DOI:** 10.3389/fimmu.2022.980709

**Published:** 2022-10-06

**Authors:** Guillaume Mestrallet, Kazuki Sone, Nina Bhardwaj

**Affiliations:** ^1^ Division of Hematology and Oncology, Hess Center for Science & Medicine, Tisch Cancer Institute, Precision Immunology Institute, Icahn School of Medicine at Mount Sinai, New York, NY, United States; ^2^ Extramural Member, Parker Institute for Cancer Immunotherapy, San Francisco, CA, United States

**Keywords:** DC, tumor microenvironment, immune checkpoint inhibitors, mregDC, Treg, cytokines

## Abstract

Dendritic cells (DCs) play a key role to modulate anti-cancer immunity in the tumor microenvironment (TME). They link innate to adaptive immunity by processing and presenting tumor antigens to T cells thereby initiating an anti-tumor response. However, subsets of DCs also induce immune-tolerance, leading to tumor immune escape. In this regard, the TME plays a major role in adversely affecting DC function. Better understanding of DC impairment mechanisms in the TME will lead to more efficient DC-targeting immunotherapy. Here, we review the different subtypes and functions of DCs in the TME, including conventional DCs, plasmacytoid DC and the newly proposed subset, mregDC. We further focus on how cancer cells modulate DCs to escape from the host’s immune-surveillance. Immune checkpoint expression, small molecule mediators, metabolites, deprivation of pro-immunogenic and release of pro-tumorigenic cytokine secretion by tumors and tumor-attracted immuno-suppressive cells inhibit DC differentiation and function. Finally, we discuss the impact of established therapies on DCs, such as immune checkpoint blockade. Creative DC-targeted therapeutic strategies will be highlighted, including cancer vaccines and cell-based therapies.

## Introduction

**Figure 1:**
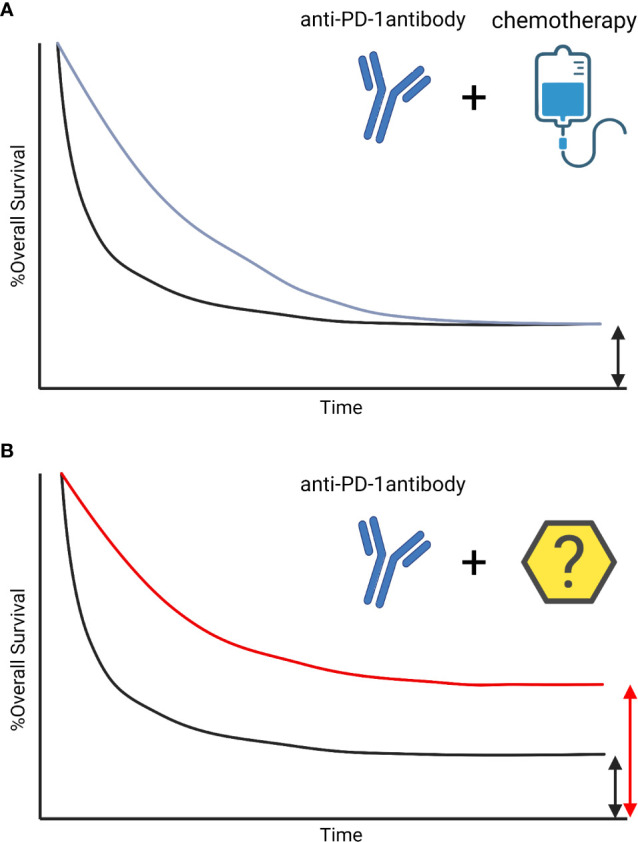
The survival curve of Immune-checkpoint inhibitors. The survival curve of Immune-checkpoint inhibitor (ICI) is characterized by patients with long-term response, called tail-plateau. The benefit of adding chemotherapies on ICIs are additive not synergistic. The combination therapies improved early-response, however, those did not lead to a sharp increase in survival after 3 years (1) **(A)**. Other treatment strategies with synergistic benefits are needed to increase the number of patients with a long-term response **(B)**.

Recent advances in immunotherapy, including the introduction of immune checkpoint inhibitors (ICI) and adoptive cell therapy, have changed the landscape of cancer care. Because of the limited response rate to ICI alone, combination therapies with other classes of drugs, including chemotherapy or molecular targeting agents, are currently being developed. ICI can elicit long-term survival, called “tail-plateau”. The combination with chemotherapy has also improved response rates and survival, however, an increase in synergistic long-term benefits compared with ICI alone was not shown (1) **(** **Figure 1** **)**. New treatment strategies are needed to increase the number of patients with long-term response. For this purpose, it is important to efficiently induce tumor antigen-specific immunity. Development of other classes of immunotherapies, such as cancer vaccines targeting neoantigens derived from genetic mutations, are making strides. These therapies enhance the tumor antigen-specific T cell response and in combination with ICI can potentially improve response rates with enhanced migration of T cells into the tumor site and epitope spreading (2). A common and important component required for the success of each of these strategies is the presentation of tumor antigens to T cells by professional antigen-presenting cells (APCs). APCs have abilities to capture, process, and present non-self-antigens to T cells, and express major histocompatibility complex (MHC) I and II as well as key costimulatory molecules for T cell engagement. Dendritic cells (DCs) are the most representative professional APCs. DCs have a unique ability to migrate to the draining lymph nodes to initiate T cell activation by presenting antigens and by providing immunomodulatory signals through cell-to-cell contacts and cytokines. 

A major issue in the development of these therapies is the impairment of DC functions in the tumor microenvironment (TME). DC function is determined by influences from surrounding cells and the microenvironment, including the TME which adversely impacts DC intratumoral entry and function, and consequently an effective anti-tumor immune response. A deeper understanding of how these DC functions are modulated and regulated by the TME will lead to the development of improved DC-targeted therapies. In this review, we will discuss the DC subtypes, functional properties and interaction with other immune cells in the TME and highlight established and developing therapies that target DC.

## Key DC functions in the TME

### DC subsets in the TME

DCs play a key role in the TME to recruit T cells and initiate the anti-tumor response in draining lymph nodes. Intratumoral DC may also play a critical role in maintaining the state of anti-tumor T cells. This lineage comprises different sub cell types that have recently been well described in the literature (3–6). The main types are cDC1, cDC2, pDC and mregDC, although other subsets such as DC3 have been alluded to. DCs are recruited into the TME by chemokines produced by NK cells, and this cross-talk along with the release of activating factors by tumor cells (e.g. RNA, DNA, RNA-DNA hybrids) causes DC activation. DCs recognize these damage-associated molecular patterns (DAMPs), mature and migrate to draining lymph nodes. There, DCs present tumor antigens to CD8+ T cells through HLA-I, initiating the anti-tumor response. These tumor-infiltrating DC (cDC1, cDC2, pDC, mregDC, MoDCs) states are conserved across solid human cancer types (7).

#### cDC1

cDC1 are CD8α+ and/or CD103+ in mice and CD141+ in humans (3). CLEC9A, CADM1 and XCR1 are additional though not exclusive markers. cDC1s are able to present tumor antigens to CD8+ T cells and to promote Th1 cell polarization of CD4+ T cells (8–12). It has been shown that basic leucine zipper transcription factor ATF-like 3 (BATF3) positive cDC1s are key factors for tumor rejection (10, 13). This is also the case for XCR1 positive DCs in mice models (14). cDC1s secrete CXCL9 and CXCL10 which facilitate the recruitment of CD8+ T cells into the TME (15). cDC1s in tumor-draining lymph nodes also maintain a reservoir of proliferative tumor-antigen specific TCF-1+ CD8+ T cells. In mice, cDC1s use CLEC9A (or DNGR-1, F-actin receptor), to recognize necrotic cell antigens (16, 17), which facilitates their uptake and processing for presentation on MHC molecules. Finally, the TME can induce the expression on cDC1 of a number of checkpoint inhibitors and inflammatory modulators such as PD-L1, ICOS, TIM3, CD39, CD137 indicating that counter-regulatory mechanisms are upregulated, potentially compromising cDC1 function (18).

#### cDC2

cDC2 are CD11c low, CD11b+ in mice and CD11c+ and CD1c+ in humans (3). They also express markers such as FcER1 and SIRP1 alpha. cDC2s stimulate CD4+ T cell responses (19, 20) including CD4+ T cell-mediated tumor immunogenicity (20). They are also able to stimulate CD8+ T cells, although not as efficiently as cDC1 in mice (21). The 2 main transcription regulators of cDC2 are T-bet and RORγt (22). A study using colon carcinoma, fibrosarcoma and melanoma cell lines injected in mice showed that type 1 IFN activates cDC2 to enhance CD8+ T cells anti-tumor immunogenicity (23). In human head and neck cancer, the presence of cDC2 may be a biomarker for survival and response to immune checkpoint blockade (ICB) (24). Moreover, the authors showed that Treg depletion relieves cDC2 suppression driving antitumor CD4+ T differentiation. However, there are some immune escape strategies developed by tumors to limit cDC2 anti-tumor activity. For example, it has been shown that CD47 expression on tumors limits tumor DNA detection by SIRPα on cDC2s (25).

#### pDCs

Plasmacytoid DCs (pDCs) are B220+ in mice and CD45RA+, CD123+ in humans and CD11c low (3). The main function of pDC is to secrete large quantities of type 1 IFNs, traditionally triggered by viral infection (5). However, the functions of pDC in the TME are still controversial as to whether they are tumor-promoting or tumor-suppressive. The analysis of TCGA data of triple negative breast cancer showed that a higher pDC gene signature is a favorable prognostic factor and is associated with IFN-γ signaling, CD8+ and CD4+ memory T cell infiltration and cytolytic activity (26). pDCs also enhance tumor antigen-specific T cell cross-priming in a NK cell dependent manner (27). On the contrary, several immunosuppressive roles of pDCs were also reported. Although pDCs promote antitumor immunity through type I IFN secretion, they can be inhibited by immunosuppressive factors such as TGFB, IL-10 and PGE2 in the TME (28). They also favor the expansion of Treg cells through ICOSL, which promotes tolerance and correlates with a poor patient prognosis (29). pDCs are also able to up-regulate the expression of indoleamine 2,3 dioxygenase (IDO), which is essential for the induction of regulatory T cells (Tregs) (27). In the TCGA data, recurrence following surgery in localized ccRCC was also associated with higher pDC and Treg infiltration (30).

#### mregDCs

Recently, it has been shown that a conserved dendritic-cell regulatory program limits antitumor immunity (31). Interrogation of the TME by several investigators has shown that mature DCs enriched in immunoregulatory molecules (referred to as mregDCs), express immunoregulatory genes (*CD274, Pdcd1lg2, IL-4R and CD200*) and maturation genes (*CD40, CCR7*). The mregDC program is expressed by cDC1s and cDC2s upon uptake of tumor antigens and suppresses anti-tumor activity in human and mouse cancers. mregDCs upregulate PD-L1, the IL-4R and down regulate IL-12. Following IL-4 blockade, the IL-12 production by tumor-antigen-bearing mregDC1s increased, favoring the expansion of tumor-infiltrating T cells and reduced tumor burden (31).

These DCs have also been referred to as DC3, although we prefer the term mregDCs as others have indicated that DC3 should refer to another subset of DC-like cells. These DC3-like cells are proinflammatory in nature and can express CD14 and CD163, produce ROS and NOS, and may play a role in Th17 induction (32–34).

### DC and T cell activation

​​Because CD8+ T cells are often the main effectors of anti-tumor immunity, promoting cross-presentation of TAAs to these cells by DCs is considered one of their most important functions. Cross-presentation of tumor associated antigens by BATF3-dependent cDC1s resulted in stronger and more effective CD8+ T cell immunity (11). WDFY4, a BEACH-domain containing protein, is also required for cross-presentation in response to tumor antigens (35). CD103+ cDC1s are recruited to the tumor site by chemokines such as CCL4 and CCL5 secreted by tumor cells. Intra-tumoral NK cells also recruit cDC1 by secreting CCL5 and XCL1, and by production of FLT3L (36).

cDC1s take up dying tumor cells and undergo maturation upon the release of DAMPs through TLRs and STING-cGAS pathways. While DCs can take up both apoptotic cells and necrotic cells, the latter lead to fruitful cross presentation and stimulation of T cells. Mature DCs expressed CCR7, which is necessary for the migration of tumor-infiltrating DCs into TDLNs where they process and load cancer antigens onto HLA-I and HLA-II for presentation to CD8+ T cells and CD4+ T cells, respectively (37). They also express co-stimulatory molecules. DC-expressed CD80 and CD86 control the activation or suppression of T cells through interaction with CD28 or CTLA4, respectively. Naive CD4 T cells are primed first and in turn license cDC1s to prime CD8+ T cells through CD40–CD40L signaling. *In vitro* DC stimulation with IL-1, IL-6, TNFα, IFNα and CD40 ligand can be used to license DC through increased expression of maturation markers and IL-12 production (38).

DCs can also produce chemokines in the TME that attract T cells. CD103+ tumor-infiltrating cDC1s are the main producers of CXCL9 and CXCL10 in the TME *via* the STING pathway, which in turn promotes the recruitment of CD8+ T cells into the TME (15). cDC1s also support T cell reactivation in the TME and may maintain stem-like CD8+ T cells (39).

The effector activity of T cells depends on DC-derived cytokines, including IL-12. In humans, both CD141+ cDC1s and CD1c+ cDC2s can produce IL-12 upon sensing IFNγ released from T cells and TLR stimulation, but IL-12 levels within human cancers are also associated with increased cDC1 infiltration (40, 41). pDC can also provide bystander activation in a TNFα- and IFNα-dependent way (42). Indeed, HIV-1-activated pDCs produced IFN-α and TNFα, migrated in response to CCL19 and matured CD11c+ DCs, which are not directly activated by HIV (42).

In summary, DCs play a central role in antitumor immunity by conditioning the TME with soluble factors, as well as attracting and mediating priming of antitumor T cells. DCs are recruited into the TME by chemokines produced by NK cells, and this cross-talk is important for DC activation.

## Modulation of DC function in the tumor microenvironment

**Figure 2:**
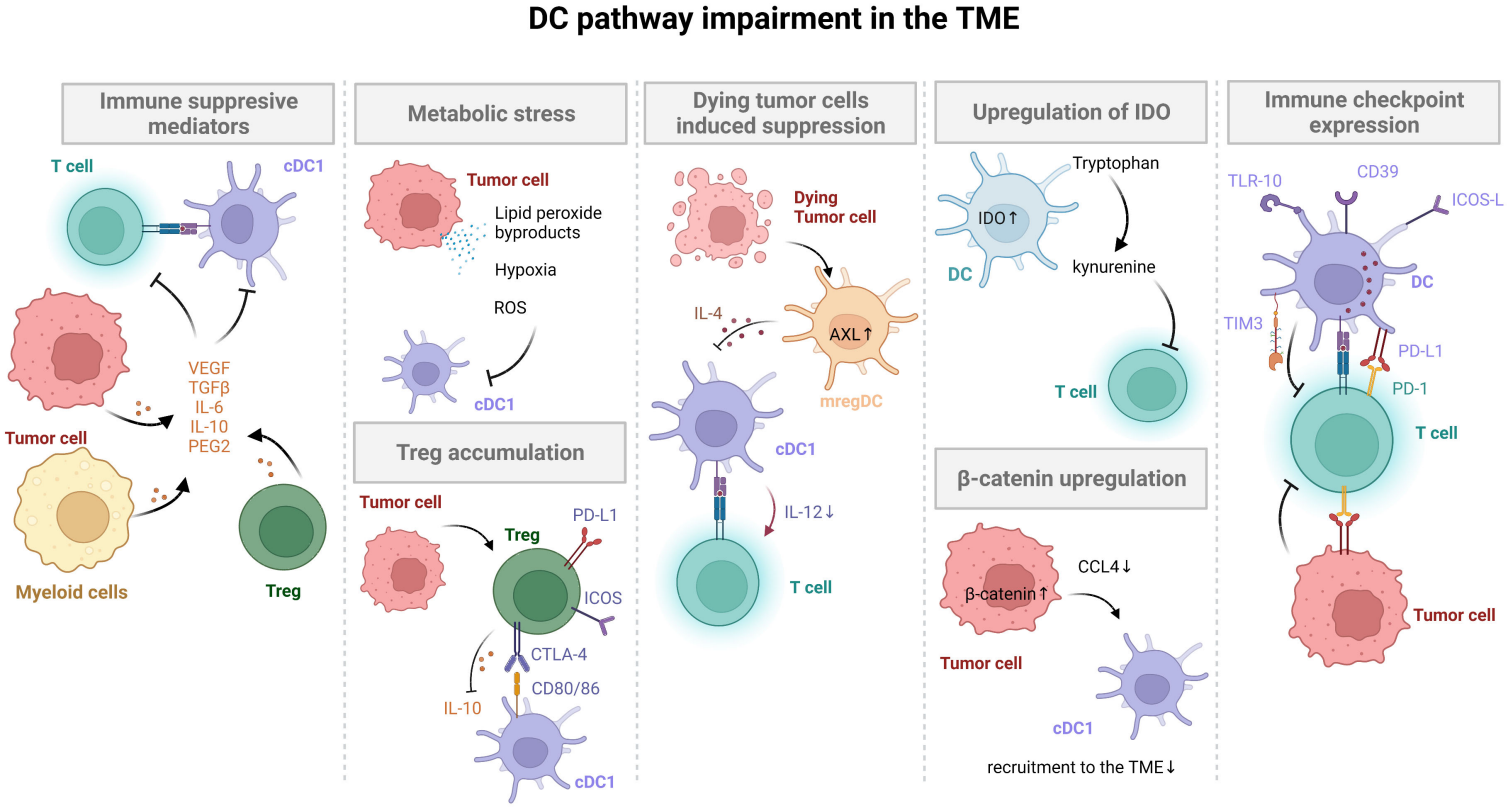
DC pathway impairment in the TME. Tumor cells inhibit DC activation and maturation through β-catenin and ROS production, soluble factor secretion (VEGF, TGFβ, IL-10, PGE2), and induction of immune checkpoint expression (PD-L1). Tumors with low neoantigen expression do not efficiently prime DCs, avoiding T cell activation. Activation of the mregDC program and additional immune checkpoint expression on DCs (e.g. TIM3) in tumors also limits T cell activation. Finally, immunomodulatory molecules secreted by tumors or expressed by DCs stimulate Treg cells, leading to tumor immune escape.

Recently, a number of studies demonstrated that the lack of spontaneous immune infiltration in solid melanoma tumors was associated with a lack of Batf3-lineage DCs using transcriptomic approaches (8, 43–46). Furthermore, failure of DC infiltration and DC impairment in the TME are key mechanisms leading to tumor immune escape for different cancers **(** **Figure 2** **)**.

### DC regulatory programs suppressing anti-cancer immunity

#### Immunosuppressive mediators

There are several pathways in the TME that adversely alter DC functions (47). Many mediators released by tumor or stromal cells have been implicated. For example, necrotic tumor cells release PGE2 that suppresses the DAMP-mediated stimulation of macrophages and DCs (48). Vascular Endothelial Growth Factor (VEGF) can also inhibit the functional maturation of DCs (49, 50). The TME production of transforming growth factor-β (TGFβ) and IL-10 block DC maturation through upregulation of the Inhibitor of Differentiation 1 (Id1) (51, 52). IL-6, a pro-inflammatory cytokine also produced by cancer cells, can inhibit DC differentiation through activation of the STAT3 pathway (53). The tumor microenvironment’s metabolic composition is also known to affect DC function. Lipid peroxide byproducts promote endoplasmic reticulum stress (ER stress) in DCs, leading to lipid accumulation in DCs (54). Lipid-accumulating DCs have reduced T cell activation and cross-presentation (55). In mouse models, oxidized lipids limit cross-presentation by sequestering the HSP70 chaperone protein and reducing MHC I–peptide translocation to the cell surface (56). Additionally, the presence of reactive oxygen species (ROS) in the TME limits DC tumor-antigen presentation (47). The ER stress sensor IRE1α can also be engaged by antigen-derived hydrophobic peptides without ER stress. IRE1α activation depletes MHC-I heavy-chain mRNAs through regulated IRE1α-dependent decay (RIDD), limiting cross-presentation. In a tumor mouse model, IRE1α disruption increased MHC-I expression on tumor-infiltrating DCs and enhanced recruitment of CD8+ T cells (57). Tumor cells also produce lactate that inhibits DC differentiation and activation (58). The transcription factors regulating DC adaptation to hypoxia are the hypoxia-inducible factors HIF1 and HIF2 (59). HIF1α enhanced DC migration and IL-22 production under hypoxic conditions (60) but limited precursor differentiation into pDCs (61). Hypoxia also downregulates the type I IFN pathway by repressing transcription and lower chromatin accessibility of STAT1 and IRF3 motifs in a HIF1/2α-independent manner (62).

#### Dead cells and TME constituents as DC mediators

DCs phagocytose both necrotic and apoptotic dying tumor cells (63). Necrotic tumor cells activate DC release of DAMPs, whereas apoptotic tumor cells can promote DC immune tolerance through different mechanisms. In cancer-free conditions, TAM family (TYRO3, AXL, MER) receptor tyrosine kinases mediate uptake and clearance of apoptotic cells and dampen inflammation, favoring homeostatic wound healing. However, the expression of these molecules on cells in the TME can lead to DC immune tolerance (64). For example, apoptotic cell uptake by cDCs leads to mregDC induction through AXL upregulation, accompanied by IL-4/13 signaling that negatively regulates IL-12 production in cDC1s, leading to immune suppression (31).

TME constituents also play a role. For example, Versican proteolysis into Versikine, degradation of IFN receptor and TLR2 activation by MMP-2 regulates DC differentiation and modulates T cell helper profiles toward a TH2 phenotype (65–67). Versikine also enhances the generation of CD103+CD11c+MHCII+ cDCs from Flt3L-mobilized primary bone marrow-derived progenitors, suggesting that Versican proteolysis may promote differentiation of tumor-seeding DC precursors toward IRF8- and BATF3-expressing cDCs in a mouse model (67).

#### Treg generation

A key point seems to be the ability of DC to contribute to the expansion and differentiation of Treg cells which limits other immune T cell activity (68). It has been shown that migratory DCs can enhance Treg generation *in vivo*, which in turn improves the outcome of experimental autoimmune encephalomyelitis in mice (69). This can be potentially done *via* several mechanisms, including PD-L1/PD-1, ICOS-L/ICOS, CD80 and CD86/CTLA-4 interactions, production of anti-inflammatory cytokines (IL-10, TGFβ, IL-27, IL-35), or expression of ILT3 and ILT4 (70, 71).

#### IDO expression

DC can express the metabolic enzyme iIDO1, especially after IFNα,β,γ exposure. This expression limits the activity of CD8+ T, NK and plasma cells and contributes to the differentiation of Treg cells through conversion of l-Tryptophan, which is an essential amino acid for T cell responses, to l-kynurenine (72). IDO1 can be stimulated by TLR signaling (71). Type 1,2 IFN, tumor necrosis factor (TNF) and TGF-β-signaling also enhance IDO1 expression (73–75). Both cDC and pDC may display this property. IDO1 is expressed in mature cDC1 but not in cDC2, but IDO1 competent cDC1 can induce regulatory cDC2 through tryptophan metabolism (76).

#### β-catenin signaling

Up-regulation of β-catenin signaling leads to a reduction of CCL4 secretion from tumor and this prevents DC recruitment into the murine melanoma TME (44). β-catenin expression was negatively associated with DC and cytotoxic T cell infiltration into the TME and associated with a poor prognosis. This was also validated in melanoma patients (77).

#### PGE2 production

In melanoma, another mechanism involves the production of PGE2 by tumor cells. PGE2 inhibits NK and DC recruitment into the TME (78, 79). Tumor production of PGE2 leads to evasion of the NK-cDC1 axis by 2 mechanisms. First, by impairing NK viability and chemokine production, and secondly by causing downregulation of chemokine receptor expression in cDC1 (79). Also, PGE2-EP2/EP4 signaling promotes inflammation by inducing expression of the NF-κB genes in myeloid cells and elicits immunosuppression by driving the mregDC-Treg axis for Treg recruitment and activation in the tumor (80).

#### Checkpoint molecule expression

Immune checkpoints are expressed on DC are also involved in DC impairment mechanisms (14). For example, tumor-infiltrating DCs suppress nucleic acid-mediated innate immune responses through interactions between T cell immunoglobulin mucin receptor 3 (TIM3) and the alarmin HMGB1 (81). The alarmin, which is released by dying tumor cells complexed to DNA, can bind to TLRs to otherwise activate DCs, an activity that is inhibited by TIM3. Moreover, programmed cell death ligand 1 (PD-L1) on DCs and TME cells inhibits proliferation and cytokine production by programmed cell death 1 (PD-1) positive T cells (82, 83). Hematopoietic progenitor kinase 1 (HPK1) is a negative regulator of dendritic cell activation (84), and is considered to be a drug target (85). HPK-1 first came to attention as a negative regulator of T cell function, namely the signaling downstream of the TCR through the AP-1, NFAT, and NFκB pathways, and reduces the expression of costimulatory molecules CD80, CD86, I-Ab and proinflammatory cytokines IL-12, IL-1β, TNF-α, and IL-6 on DCs required for effective anti-tumor immunity (84–86). cDC1s also express other checkpoint molecules and T cell agonists including LAG3, CD200A and GITR, ICOS(L), LAG3, OX40L, respectively, in addition to the immunomodulatory molecules BTLA, TLR10 and CD39 (18).

### Inhibition of DC function in the TME: Effects on tumor immunogenicity

Tumor cells modulate DC function in several ways as discussed above. It is also important to understand how the mechanisms of DC suppression differ according to the immune profile of the TME in order to develop more targeted therapies. Immunogenic tumors are often characterized by high mutation burden and this can be associated with enhanced infiltration by T cells, DC, M1 macrophage polarization and increased expression of other immune-associated genes (87).

Although an immunogenic TME is elicited by DC and in turn positively impacts DC function, DC function can be potentially affected through adaptive resistance mechanisms. For example, microsatellite instability-high (MSI-H) tumors which include colorectal, gastric and endometrial cancers, are characterized by loss-of-function mutations in one allele of the genes for MLH1, MSH2, PMS2, MLH6, and EPCAM (88). In sporadic and inherited cancers (e.g. Lynch syndrome, LS) there are loss of function mutations or hypermethylation of genes expressing MLH1, MSH2, MSH6, PMS2 and EPCA (84, 85). This leads to a higher frequency of insertion and deletion events that take place in microsatellite regions of the genome, which accompany oncogenic driver mutations (89). Unstable DNA intermediates lead to STING activation and the production of type I IFNs that promote immunity (90). It has been shown that DC infiltration in colorectal cancer (CRC) correlated with other tumor-infiltrating CD4+ and CD8+ T cells (91) and that LS patients had elevated mucosal T-cell infiltration even in the absence of cancer (92). However, advanced CRC can become resistant to ICI, and can be characterized by lower levels of CD83+ DC infiltration in the colon tumor stroma (93). Moreover, the frequency of distant metastases was higher in patients who had lower DC numbers. These patients also had a shorter overall survival. Thus, DC infiltration may be essential for T cell priming and infiltration and consequential for MSI-H tumor regression.

High numbers of tumor-infiltrating Foxp3-positive Treg cells were also detected in MSI_H tumors that also showed a low proportion of mature DC (94). The correlation of Foxp3-positive Treg cell density with low levels of mature DC suggested that impaired DC maturation may contribute to immune escape in CRC. Another study in CRC patients showed that polyclonal Treg expansion limited DC function and anti-tumor immunity (95). Immune-checkpoint molecules may also be involved in DC regulation. The number of PD-L1 positive DCs in the TME correlated with CD8 infiltration in CRC (96). Indeed, PD-L1 expression on DC is upregulated by inflammatory cytokines which are rich in immunogenic TMEs (97). PD-L1 may also participate in the suppression of DC-mediated T cell activation. Interestingly, oncogenic signaling also impairs DCs in the TME with high neoantigen loads. In non-small cell lung cancers (NSCLC) with high neoantigen load, β-catenin expression was associated with low levels of CCL4 in the TME, resulting in reduced DC infiltration into the tumor (98, 99). Moreover, in melanoma animal models, this reduction in CCL4 impairs DC recruitment and resulting anti-tumor immunity (44). In MSS CRC, which has a much less inflammatory TME than MSI-H CRC, it was reported that the tumors can express neoantigens with high predicted HLA-I affinity, but these were broadly expressed at lower levels compared to those from MSI-H CRC. MSS primary CRC have a paucity of dendritic cells which potentially limits cross-presentation and thus may contribute to the T cell dysfunction observed (100). Additionally, in mismatch repair-proficient colorectal cancer liver metastases, a paucity of DC (and of activated T cells) limited immune checkpoint blockade efficacy (101). Dendritic cell mobilization and recruitment and stimulation by Flt3L, IFNα and/or local radiation therapy improved ICB efficacy in a mouse model (101).

## Overcoming strategies to tackle DC impairments in the TME

**Figure 3:**
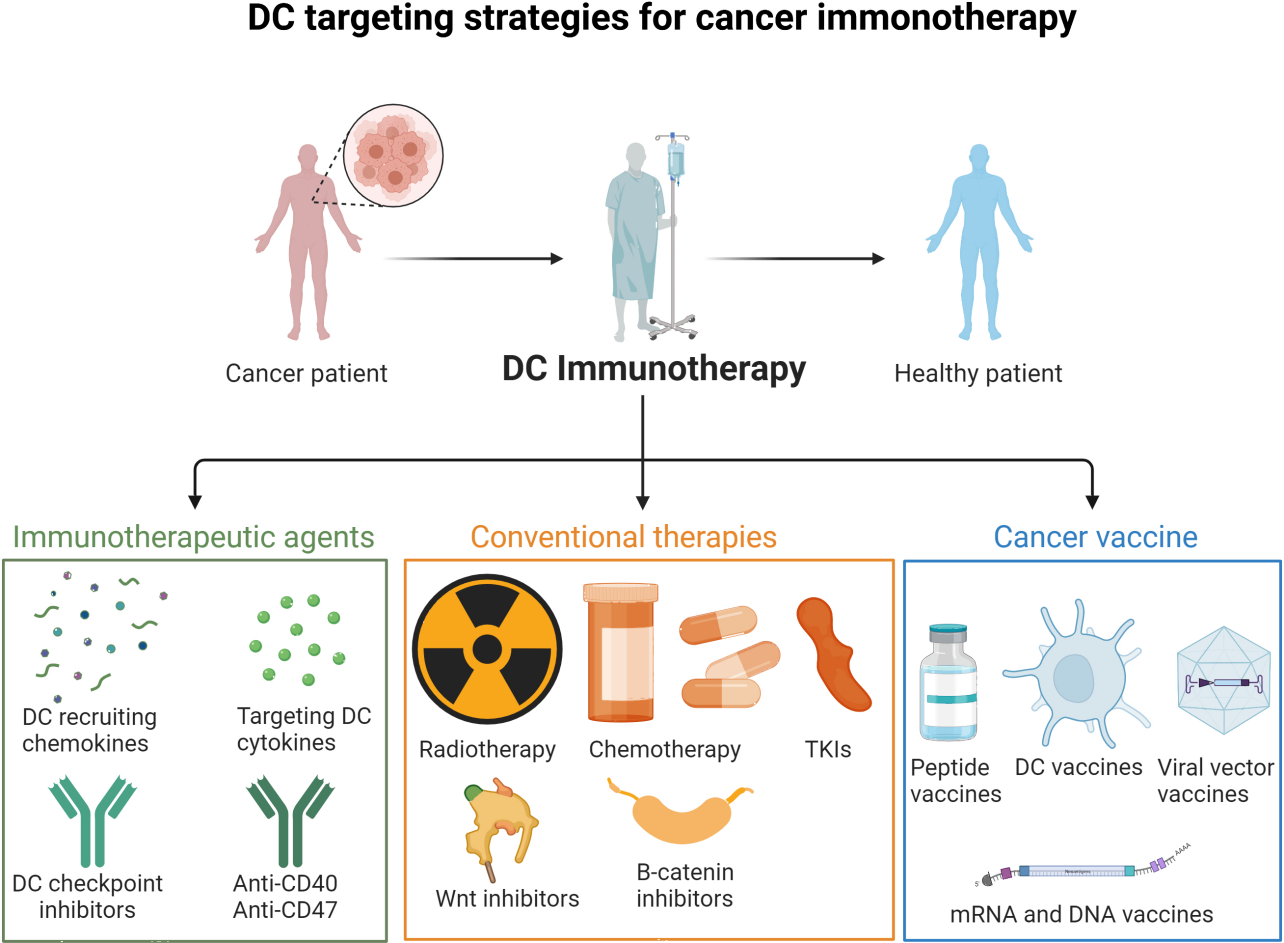
DC targeting strategies for cancer immunotherapy. Strategies to overcome DC impairment and recruitment in the TME include immunotherapeutic agents (DC checkpoint inhibitors, DC recruiting cytokines and mobilizing agents), conventional therapies (radiotherapy, chemotherapy, TKIs, Wnt, B-catenin and inflammasome inhibitors), and cancer vaccines (mRNA, DNA, peptide and DC vaccines).

DC recruitment/impairment of function in the TME seem to be key issues favoring the tumor immune escape. Thus, it is of interest to develop therapeutic strategies to tackle tumor-induced DC dysfunction or a paucity of tumor infiltrating DCs (102) **(** **Figure 3** **,** **Table 1** **)**.

**Table 1:** Overcoming strategies to tackle DC impairments in the TME.

Targets of therapy	Mechanisms	Examples of treatment modalities
Targeting DC immune Tolerance mechanisms in the TME	Upregulation of immune-checkpoint molecules	PD-1/PD-L1 blockade
		TIM-3 blockade (activates inflammasome on DC)
		LAG-3 blockade
	Dead cell mediated immune tolerance program	Anti-IL4/13 antibody (mreg DC) TAM (TYRO3, AXL, MER) family inhibitors
	IDO1 expression	IDO1 inhibitor
		IDO1/PD-L1 Immunomodulatory vaccine
Inhibiting immunomodulatory small molecules	VEGF/VEGFR	Anti-VEGF/VEGFR antibody Multi-kinase inhibitor (cabozantinib etc.)
	PGE2	PGE2 inhibitor
	Other cytokines	Anti-IL-1β antibody or anti-IL-6 antibody etc.
Overcoming DC suppression by cancer cells	Accumulation of Treg cells	Anti-CTLA-4 antibody, anti-VEGFR2 antibody
		Treg targeting drugs: - chemokine receptors (CCR4 or CCR8) - immune-checkpoint and T cell agonists (OX40, GITR, ICOS) - immunosuppressive molecules (CD39, PI3Kδ etc.)
	Upregulation of Wnt/β-catenin signaling in cancer cells	m-TOR inihibitor/multi-kinase inhibitor
		Selective Wnt/β-catenin inhibitor
	Inactivation of dendritic cells in low immunogenic tumor	Immunogenic cell death inducers (chemotherapy or radiotherapy etc.)
		Adjuvants (TLR agonist etc.) DC-mobilizing agents (FLT3L etc.) DC-specific antibodies inducing maturation or antigen uptake (CD40 agonistic antibody, Clec9A etc.)
**Expected novel DC-targeted strategies**		
Cancer vaccine	DC targeting vaccines	Personalized neoantigen peptide with adjuvant
		Delivery with DC-specific antibody (DEC-205, CLEC9A)
		Delivery with DC mobilizing agents (Flt3L etc.)
		Delivery route (e.g. *in situ* vaccination, intravenous nanoparticle vaccine)
		mRNA, DNA, viral based vaccines
	DC vaccine	moDC or natural DC subset (e.g. cDC1)
		TAA, neoantigen or whole tumor lysate loading
		Delivery route e.g. IV or intratumoral DC vaccination
		Gene-modified DC

### Targeting immune tolerance pathways in DCs

#### CD40 ligation

Leveraging approaches to overcome DC dysfunction, ineffective maturation and failure of recruitment are designed to reverse “tolerance” in tumor associated DC. For example, single-cell analysis in a CRC study highlighted that treatment with anti-CD40 activated DCs and increased CD8+ memory T cells (103). Anti-CD40 agonistic antibody has also been used in pancreatic cancer with or without chemotherapy and anti-PD-1 antibody or in neoadjuvant settings (104, 105). These agents were tolerable and led to DC maturation and T cell infiltration in the tumor microenvironment.

#### PD-1/PD-L1 blockade

Systemic PD-1/PD-L1 blockade improves the interactions between not only T cells and tumors.

The efficacy of anti-PD-1/PD-L1 antibodies has been demonstrated in many types of cancer, including MSI-H cancer (83, 106, 107). These treatments also positively affect DC-mediated anti-tumor immunity. It has been shown that PD-1 and PD-L1 expression on DCs suppresses CD8+ T cell function and antitumor immunity, indicating that these checkpoint molecules impact DC function (83, 108, 109). cDC1 upregulate PD-L1 upon antigen uptake following IFN-γ stimulation (83). Deletion of PD-L1 in DCs limited tumor growth and increased antitumor CD8+ T-cell responses, emphasizing the importance of DCs in the regulation of T cell immunity in cancer (108). PD-1 is also expressed on DC subsets. PD-1-deficient DCs secrete more IL-2 and IFN-γ and have a superior ability to stimulate antigen-specific CD8+ T cells (109). In 2022, FDA approved anti-PD-1/PD-L1 antibody treatments in 22 cancers with or without other checkpoint inhibitors, small molecule-targeted therapies, chemotherapy and so on. Recently, the FDA approved the first combination therapy targeting LAG-3 and PD-1 for melanoma (110). LAG-3 is also involved in DC immune suppression. LAG-3 deficiency has led to increased TNFα secretion and upregulation of glycolysis in murine bone marrow derived DC (111).

#### TIM3 blockade

T cell immunoglobulin and mucin-containing molecule 3 (TIM3) is an immune checkpoint expressed on IFNγ producing T cells (112). Using single-cell RNA sequencing, it was shown that loss of TIM3 on DCs promotes anti-tumor immunity through increasing the accumulation of ROS, leading to inflammasome activation (113). These results were confirmed by other studies in mouse models showing that TIM3 on DCs downregulates the cGAS-STING pathway by suppressing extracellular DNA uptake (114). Finally, TIM3 regulated cDC1 function and response to chemotherapy in breast cancer in a mouse model (115). Several Phase I studies using a combination of TIM3 and PD-1/PD-L1 antibodies have been conducted with promising results but these will require confirmation in larger studies (116–118).

#### Targeting apoptotic and mregDC tolerance programs

The recent discovery of the mregDC program revealed cDC1 and cDC2 potential impairment mechanisms in human and mouse cancers (31). Blocking IL-4 enhanced IL-12 production by tumor mregDC1s and expanded the pool of tumor-infiltrating T cells. Importantly, it also reduced the tumor burden. IL-4, but also the IL-13 pathways, were previously shown to promote tumor growth (119). Thus, targeting these pathways is of interest to overcome DC impairment in cancer. An anti-IL-4/IL-13 antibody is approved for severe asthma (120). This antibody is being tested in combination with an anti-PD-1 antibody in lung cancer (NCT05013450). Apoptotic cell uptake by cDCs may also lead to mregDC induction through AXL upregulation. Small molecule kinase inhibitors of apoptotic cell capturing molecules of the TAM family are being evaluated in the clinic, with evidence of efficacy (121).

#### Targeting soluble factors (VEGF, PGE2 and other cytokines/mediators)

DC can be targeted *via* inhibitors of soluble factors that down-modulate DC function. It has been shown that the VEGF inhibits the functional maturation of DCs (49) including VEGF produced by human breast and colon adenocarcinoma cell lines (50). Anti-VEGFR antibodies reduce Treg activation in the TME, which may further promote DC activation (122). Even if these approaches are not novel, they have been clinically tested in cancers such as lung cancer (123). However, the overall complementary effect of angiogenesis inhibitors with immune checkpoint inhibitors is not seemingly significant, and further biomarker exploration is expected to identify candidates that may be more responsive. In RCC, ICI showed promising results (124), and Cabozantinib, a potent inhibitor of VEGF, AXL and MET receptors, has also been approved for first line use with Nivolumab in patients with advanced RCC (125). There is also an ongoing clinical trial involving the inhibition of PD-1 and VEGF in microsatellite-stable endometrial cancer (126). PGE2 inhibitors, such as celecoxib, are currently under development in pre-clinical models (127–129). Inhibition of several tumor immunosuppressive cytokines blocking the inflammasome pathway are also in the clinic or under development, such as anti-IL-1bR, anti-IL-1, IRAK4 (130, 131) and IL-6 inhibitors (132).

#### IDO1 inhibition

IDO1 (Indoleamine-pyrrole 2,3-dioxygenase) is one of the enzymes that catalyzes L-tryptophan to N-formylkynurenine. It is also expressed by DCs and regulated by IFNs(α,β,γ) (72). Importantly, kynurenine production is toxic for CD8+ T, NK and plasma cells but favors Treg cell differentiation. IDO1 inhibitors were tested in several clinical trials for cancer immunotherapy (133, 134). However, they did not improve progression-free survival or overall survival. Recently, the results of a combination study using an immunomodulatory vaccine against IDO1/PD-L1 with an anti-PD-1 antibody (nivolumab) showed promising results with an 80% response rate including 43% complete responses in patients with metastatic melanoma (135). After a follow-up of 23 months, the median progression-free survival was 26 months, even if the median overall survival was not reached. CD4+ and CD8+ T cells with activity against IDO1+ and PD-L1+ cancer and immune cells were detected in the blood of the vaccinated patients.

### Increasing DC activation to counter tumor immunomodulation

Tregs have DC inhibition properties and accumulate in highly immunogenic tumors, through chemokines such as CCL17/22 (136). In a mouse model, it was shown that Treg depletion relieves cDC2 suppression thereby driving antitumor CD4+ T differentiation (18). Anti-CTLA4 antibodies cause Treg depletion effects in mouse models (137, 138), but not in humans. Other therapies targeting chemokine receptors on Tregs, such as CC Chemokine Receptor 4 (CCR4), which is expressed on Tregs (and Th2 cells) are under evaluation e.g. mogamulizumab (139). Several other Treg-targeted therapies are in development, including metabolic adaptation targeted therapies (136). Another strategy has been to inhibit the β-catenin pathway to increase DC migration to the TME through upregulation of CCL4 secretion, directly (140) or with mTOR inhibitors and tyrosine kinase inhibitors (TKIs) (sorafenib, sunitinib) (3). Temsirolimus is a mTOR inhibitor that enhances the efficacy of DC vaccination (141).

Induction of activated mature DCs is important in “COLD” or poorly immunogenic tumors with low mutational or neo-antigen expression. To engage DCs, tumor cell death induced by chemotherapy and radiotherapy have been deployed (142). DCs are activated through DAMPs, such as HMGB1 or ATP, released as a result and by other immunogenic cell death inducers such as oncolytic virotherapy and photodynamic therapy. In several cancers, combination therapy using ICB and chemotherapy have had good responses even for cancers with low PD-L1 expression (143). Many studies have been conducted in lung cancer in this regard, showing that the addition of local radiation to ICI therapy improved response (144). Maintenance therapy with anti-PD-L1 antibody after chemoradiation therapy has also been successful (145).

Adjuvants that stimulate DCs, such as TLR agonists, is another approach to activate tumor associated DC. TLR3 agonists targeting CD141+ cDC1 are particularly representative (146). CD40 agonists that promote DC maturation are also in clinical development (147) (see **Figure 3**). Recently, a phase II trial (NCT02129075) showed that fms-like tyrosine kinase 3 (Flt3) ligand pre-treatment enhanced responses to dendritic cell (DC)-targeting vaccines in melanoma patients (148). Tumors are also known to create an immunosuppressive environment by controlling the metabolic conditions. Metabolomics modulation may be combined with other strategies to better tackle DC impairment in cancer (149).

### Novel DC-targeted strategies

#### Cancer vaccine and cell therapy

Recently, technological advances have led to the development of vaccines targeting personalized neoantigens (150). In addition to simple antigen based vaccine injections, therapies that aim to increase the efficiency of antigen-specific immune induction and reduce adverse reactions are also being developed (151). Targeting delivery of antigens and adjuvants to DCs using DC-specific antibodies can increase the efficacy of vaccination. Anti-DEC-205 antibodies or anti-CLEC9A antibodies display enhanced cross-priming activity when conjugated with antigen (39, 152), and the former has been successfully tested in the clinic showing induction of anti-TAA immunity (148, 153). It is also possible to target XCR1 at the surface of human cDC1 to specifically induce CD8+ T cell responses (154). The addition of DC-mobilizing agents, such as Flt3 to these treatments enhance vaccine efficacy in humans (155). Combination adjuvants including poly-ICLC that target DC *in vivo* also improve vaccine efficacy (148). The route of vaccination may further enhance vaccine efficacy. *In situ* vaccination in non-Hodgkin lymphoma patients combining FLt3L, polyICLC in addition to radiation, enhanced the efficacy of checkpoint blockade (156). Intravenous-self assembling nanoparticle cancer vaccines that contain a TLR7 agonist induce a higher proportion of TCF-1+ PD-1+ stem-like T cells as compared to subcutaneous immunization. However, subcutaneous vaccines generate more T cells enriched in effector genes (151). mRNA-based vaccines and methods to deliver mRNA into DCs are also under development and have been used in neoantigen vaccine trials and are being evaluated in phase II and III trials (NCT03815058, NCT03897881 and so on) (157). mRNA-based vaccines have several advantages, such as the high potential for rapid development, low-cost manufacture, DC activating potential and safe administration (2). These vaccines which have incorporated personalized neoantigens, may potentially prolong time to recurrence. Incorporating shared neoantigens into such types of platforms e.g. tumor-specific antigens derived from shared frameshift mutations in MSI-H cancer and Lynch syndrome patients, may be sounds approaches to develop common “off-the-shelf” cancer preventable vaccines for patients with MSI-H cancers or Lynch syndrome (158).

DC vaccines to treat cancer have been evaluated in hundreds of trials (88, 159, 160). Only one DC-based vaccine has been approved for castrate-resistant prostate cancer, although modest in its effect in castrate resistant prostate cancer (161). DC vaccines have so far mainly used moDC differentiated from CD14+ monocytes and CD34+ progenitors *in vitro*, and a variety of antigens. Neoantigen-loaded moDCs have proven to be immunogenic in melanoma patients inducing CD8+ T cells (161). Whole tumor lysate-loaded DC vaccines also enhance antigen-specific immune responses and induce anti-cancer effects in several cancers including renal cell carcinoma, melanoma and glioblastoma (162). Gene-modified or metabolically labeled DC vaccines can increase chemokines or cytokines in the TME and increase the efficiency of antigen-specific T cell induction (163, 164). DC vaccines could also be used in combination with other modalities, such as chemotherapeutic agents. Indeed, these agents stimulate and activate DCs to promote immunity against human CRC cells through upregulation of the transporter associated with antigen processing (165). Intratumoral DC vaccination has also been evaluated (166). Naturally occurring DCs *in vivo*, such as cDC1, are more capable of inducing antigen-specific immunity than moDCs (4, 167). Indeed, the loss of cDC1 prevents effective anti-tumor immunity which can be restored upon cDC1 intratumoral injection (168).

As there are only 0.02% cDC1s in the blood, there is a need to generate these DCs *in vitro* to test their functionality *in vivo*. Recently, protocols to do so have been designed and we await the application of this approach to the clinic (169). Clinical application of CAR-T cells is progressing, especially in hematologic malignancies. However, tumor antigen heterogeneity remains a challenge limiting their efficacy against solid cancers. To address this, T cells were engineered to secrete the DC growth factor Flt3L. Flt3L-secreting T cells expanded intratumoral cDC1s and increased host DC and T cell activation when combined with immune agonists poly (I:C) and agonistic anti-4-1BB, leading to enhanced tumor growth inhibition (170).

## Conclusion and perspectives

Immunotherapies have changed the treatment and clinical outcomes of cancer patients but immune resistance affects success rates. It has become evident that even DCs are subject to immune dysregulation and factor as one of the etiologies of immune resistance. The efficacy of immune checkpoint inhibitors has been established for “Hot” tumors, but dissecting the immune escape mechanisms targeting DCs may make them more effective. There is also potential in this area to extend vaccine therapy to a prevention approach. For “Cold” tumors, it is necessary to develop a more comprehensive strategy, including improving DC infiltration into the tumor site, vaccines and ACTs in addition to conventional chemotherapy and radiotherapy. Recognizing the immunological characteristics of individual patients and developing a well-defined therapeutic strategy will further personalized precision medicine. For this purpose, it will be necessary to establish a more accurate and simple evaluation system for enumerating DC in the TME and determining how and when they are specifically modulated within the TME.

## Author contributions

GM and KS wrote the first draft of the manuscript. NB revised the manuscript and acknowledged the final version. All authors contributed to manuscript revision, read, and approved the submitted version.

## Conflict of interest

The authors declare that the research was conducted in the absence of any commercial or financial relationships that could be construed as a potential conflict of interest.

## Publisher’s note

All claims expressed in this article are solely those of the authors and do not necessarily represent those of their affiliated organizations, or those of the publisher, the editors and the reviewers. Any product that may be evaluated in this article, or claim that may be made by its manufacturer, is not guaranteed or endorsed by the publisher.
